# ERRATUM: Nursing Activities Score: an updated guideline for its application in the Intensive Care Unit

**DOI:** 10.1590/1980-220X-REEUSP-2024-ER18en

**Published:** 2024-10-04

In the article “Nursing Activities Score: an updated guideline for its application in the Intensive Care Unit” , with the DOI: https://doi.org/10.1590/S0080-623420150000700019, published by the journal: “Revista da Escola de Enfermagem da USP, volume 49 (spe) of 2015, p. 131–137, on page 137:

**Now read:**

## Financial support

This study was financed in part by the Coordenação de Aperfeiçoamento de Pessoa de Nível Superior – Brasil (CAPES) – Finance Code 001

